# Co_2_ Laser Resurfacing for Facial Rhytides

**DOI:** 10.4103/0974-2077.41152

**Published:** 2008-01

**Authors:** Vinod K Jain, B C Ghiya, Dhruv Gupta, Mahendra K Singhi

**Affiliations:** *Department of Skin and VD, SN Medical College, Jodhpur, Rajasthan, India*

**Keywords:** Carbon dioxide laser, facial rhytides, resurfacing

## Abstract

Resurfacing of facial rhytides (periorbital crow-feet wrinkles) was performed in three cases by carbon dioxide laser (Sharplan^®^ 1030 machine). Good to excellent results were observed. However, erythema and postinflammatory pigmentation were important side effects.

## INTRODUCTION

Resurfacing denotes mechanical, chemical or physical removal of superficial layers of ageing skin stimulating neocollagenosis and improvement in epidermal architecture.[1] In order to treat wrinkles, it is necessary to resurface up to the papillary dermis and sometimes up to reticular dermis so that old collagen is replaced by new collagen, resulting in tightening of skin.[2]

Facial rhytides is a common cosmetic concern that can be treated successfully with lasers.[3] There are various types of lasers available to treat facial rhytides *viz*, carbondioxide (CO_2_) laser, Erbium-YAG laser, etc. These lasers typically restrict the laser tissue interaction time to <1 ms (thermal relaxation time, TRT) so that thermal diffusion is limited during the laser pulse. CO_2_ laser resurfacing is a technique for the treatment of skin ageing. With this it is possible to perform accurate ablation of skin layer by layer. Pulsed CO_2_ laser is a modality, which has an ability to do resurfacing with safety, giving excellent cosmetic result. In the present article, we share our experience of CO_2_ laser in three cases with facial rhytides.

## MATERIALS AND METHODS

Three patients of periorbital rhytides between ages of 35 and 40 years were selected. Of them, two patients had type IV skin and one had type III skin. All three patients were females. They were informed verbally about the procedure, possible outcome and complication and detailed written consent was obtained. Preoperatively tretinoin 0.05% and hydroquinone 2% once daily for 3 weeks were applied. Oral acyclovir was given 2 days before resurfacing.

We used a super pulse CO_2_ (SHARPLAN^®^ 1030) by setting power at 1 W in defocused mode. After giving local anaesthesia, the surrounding area was covered with wet gauze. Two passes were carried out. Passes were made at the shoulder of wrinkles. There was skin contraction after second pass. Yellowish surface appeared after two passes. Few deep wrinkles required three passes. The treated area was wiped with the gauze soaked in hydrogen peroxide after each pass. There was no bleeding after treatment. Post-operative dressing was done with Sofratulle^®^, which was changed after 2 days. Then the treatment area was kept open and patients were asked to apply mupirocin ointment twice daily after cleansing with mild cleanser. Sun exposure was strictly restricted for at least 2 weeks.

All patients were followed at 1 week, 2 weeks, 2 months and 6 months after the procedure. At each follow-up visit, photographs were taken and apparent improvement in wrinkles, the presence or absence of erythema, infection, dyschromias and scarring were noted. Improvement was graded on a scale of 1-5, where score of 1 indicated poor, 2 - fair, 3 - good, 4 - excellent, 5 - complete clearance or 100% resolution of wrinkles.

## RESULTS

Of the three patients treated, two patients completed the follow-up of 6 months, but one patient was lost to follow-up after 15 days.

All three patients developed erythema during second week, but it subsided in two patients, one patient did not come for follow-up. All patients developed edema after treatment, but it subsided within 2 days. Both patients developed hyperpigmentation, which subsided within 6 months in one patient and after 2 months in the other patient. It was treated by mild steroid and 2% hydroquinone cream. There was no incidence of infection, hypopigmentation or scarring.

The two patients who were available for follow-up, showed grade 4 or excellent improvement and expressed satisfaction at the results.

## DISCUSSION

For many years, dermabrasion, chemical peeling or surgical face lifting have been used for the treatment of facial wrinkles. However, CO_2_ laser which emit ultra short, coherent light beam enables the cosmetic surgeon to ablate superficial epidermis layer by layer with no or very little side effects.[4] Many modalities are available for the treatment of facial, especially periorbital wrinkles like topical tretinoin creams, glycolic acid chemical peel, botulinum toxin, collagen filler, dermabrasion and laser resurfacing.[5] Previous treatment modalities such as dermabrasion have been limited by the complications of scarring and pigmentary alteration. With the advent of high energy, pulsed CO_2_ laser, skin resurfacing can now be done successfully with minimal side effects.[6] However, deeper wrinkles of glabella and nasolabial fold respond best to classical face lift. Beside wrinkles and pigmentary changes of ageing skin, the CO_2_ laser has been found to be useful in all types of scar namely, atrophic, hypertrophic, scars of varicella, etc.[7]

In our study, excellent results were achieved in two patients available for follow-up. However, the post-procedure pigmentation was a concern though it subsided completely in both the patients.
